# The Effectiveness of Internet-Based Cognitive Behavioral Therapy in Treatment of Psychiatric Disorders

**DOI:** 10.7759/cureus.1626

**Published:** 2017-08-29

**Authors:** Vikram Kumar, Yasar Sattar, Anan Bseiso, Sara Khan, Ian H Rutkofsky

**Affiliations:** 1 California Institute of Behavioral Neurosciences and Psychology, Sri ramachandra University; 2 Research Assistant Psychiatry, SUNY Downstate University; 3 College of Medicine, Al-Quds University; 4 California Institute of Behavioral Neurosciences and Psychology, Dow Medical College, Pakistan; 5 Research, California Institute of Behavioral Neurosciences & Psychology

**Keywords:** internet, web-based interventions, mobile-based psychotherapy, cognitive behavioral therapy, e-mental health, depression, self help

## Abstract

This review article is an overview of the effectiveness of internet-based cognitive behavioral therapy (ICBT) in the treatment of psychiatric disorders. ICBT’s effectiveness has been investigated in treating and managing conditions like depression, generalized anxiety disorder (GAD), panic disorder, obsessive compulsive disorder (OCD), post-traumatic stress disorder (PTSD), adjustment disorder, bipolar disorder, chronic pain, and phobias. ICBT’s role in the treatment of medical conditions such as diabetes mellitus with comorbid psychiatric illnesses was also explored. Furthermore, this study elaborates on its cost-effectiveness and its impact in rural areas. We conducted a thorough literature search using PubMed and Google Scholar with no restrictions on the date. ICBT's role in treating and controlling psychiatric illnesses has been established in the literature. From the data compiled, we conclude that ICBT is useful in treating mental health and medical illnesses with psychiatric comorbidities. It has also been found to be cost-effective for patients and society. ICBT is a potential tool emerging with modern day technological advancements and is useful in rural and urban settings, across various languages and cultures, and on a global scale. Larger randomized control trials on its use in clinical practice and in reaching rural populations are bound to shed more light on the effectiveness of this tool along with spreading awareness among physician and patient communities.

## Introduction and background

“An 18-year-old female presented to the emergency room of a hospital in Chicago, Illinois, with complaints of palpitations, sweating, intense fear, shortness of breath, generalized numbness, and a feeling that something 'terrible was going to happen'. She has had similar episodes previously and often worries about their recurrence. After running all the prerequisite tests, she was diagnosed with panic disorder. The patient refused pharmacological treatment and did not appear interested in outpatient therapy. While being discharged, she mentioned that her friend also suffers from anxiety and recently started 'internet therapy' on her mobile phone, and she wanted to know more about it.”

This is a typical example of a patient receiving psychotherapy through the internet. Internet-based cognitive behavioral therapy (ICBT) is therapy provided through a computer or a mobile device. With technological advances and its integration with healthcare, it has become a fast-growing intervention channel compared to conventional psychotherapy. ICBT is used to manage various psychiatric disorders such as mild to moderate depression and other disorders such as anxiety, obsessive compulsive disorder (OCD), and panic disorder [1-3]. ICBT has also been used to treat patients who have chronic illnesses such as diabetes with comorbidities such as depression and in those who received intensive care and had developed post-traumatic stress disorder (PTSD) [4-5]. Traditional therapies face drawbacks, including cost inefficiency and a lack of follow-up, especially among younger adults and patients belonging to a low socio-economic status, but ICBT has been shown to be more cost-effective in this context [6-7]. ICBT has also been shown to increase the functionality of patients and decrease symptoms and severity [8]. In the case of therapist-guided ICBT, it has shown high user satisfaction and an overall decrease in symptoms [9].

In this article, we concentrate on the growing potential of ICBT in the management of various psychiatric disorders and its efficacy in patient care. Therapist-guided ICBT involves checking in with the therapist via emails or weekly online sessions [1]. Self-guided ICBT requires the patient to use an app or an internet program such as 'MoodHacker' or 'e-Ouch' [8]. These programs are used to treat mild to moderate depression and for pain management. Applications like Twitter can be utilized for patient coaching and can link with the ICBT app easily for sharing posts [10-11]. There are also web pages like 'Living Life To The Full,' which deliver cognitive behavioral therapy (CBT) for maladaptive thoughts, sleeping disorders, anxiety control, relaxation techniques, exercise, and diet.

This review article explores the effectiveness of ICBT in the treatment of psychiatric disorders. First, it establishes the components of ICBT and explores its origin and function. Next, the study investigates the use of ICBT in treating medical illnesses with psychiatric comorbidities. Its cost effectiveness has also been discussed, in terms of both patient costs and societal costs. Moreover, ICBT’s impact in rural areas is examined. Literature shows that rural areas are prone to lower rates of patient follow-up and lack resources. The aim is to establish ICBT’s effectiveness in such locations. However, there is a need for further larger scale studies on ICBT. The day-to-day effectiveness of ICBT in clinical practice would have to be documented by such research. Moreover, the evidence has also highlighted the need for more research on the impact of ICBT in rural and low socioeconomic areas, and has shown the benefits derived from its use in such places.

## Review

This review was conducted using relevant publications about internet-based, web-based, and mobile-based cognitive behavioral therapy indexed in PubMed, PubMed Central (PMC), and Google Scholar. A thorough review of the references revealed further relevant articles. This article focusses on the use of ICBT in the treatment of psychiatric disorders and medical conditions with comorbid psychiatric disorders. Studies that appeared to be biased and which misconstrued data were excluded. In total, 373 articles were reviewed from PubMed with no date limitations, and articles on ICBT from Google Scholar and PMC were included. These were the following keywords searched: internet-based, web-based, mobile-based, internet, app, cognitive behavioral therapy, depression, anxiety, obsessive compulsive disorder (OCD), post traumatic stress disorder (PTSD), generalized anxiety disorder (GAD), panic disorder, psychotherapy, psychiatry, mental health, e-mental health, mood disorder, e-medicine, technology, self-help, and addiction.

Internet-based cognitive behavioral therapy (ICBT): origin and structure

ICBT was first practiced in the late nineties when researchers across the globe were exploring internet-based psychotherapy treatments [12]. In the book, “Guided Internet-Based Treatments in Psychiatry,” edited by Linderfors and Andersson, three indispensable parts of ICBT were identified: "secure electronic treatment platform, proper treatment program, clinician guidance” [12]. The electronic platform is an internet-based, computer or smart phone compatible software in the form of an app or web page. One such application is MoodGYM, which was developed in Australia to provide ICBT to patients with depression and anxiety [13]. It requires the user to set up a personalised username and password, ensuring data protection. These apps and web pages can be produced in multiple languages, ensuring dissemination on a large scale [13].

The duration and type of treatment program offered are of equal importance. Studies report different lengths of programs. For instance, while some simplified ICBT programs are designed to last for as short as five minutes [14], other programs, such as a randomized control trial on ICBT in panic disorder, lasted for 12 weeks [15]. The patient completes assignments such as a sleep diary, recording maladaptive thoughts, and participating in relaxation exercises, with or without therapist support [16-17]. Patients also have options to check in with the therapist in person; however, studies show that ICBT is as effective as face-to-face therapy [18]. Furthermore, ICBT is useful in treating psychiatric conditions which are comorbidities of underlying organic diseases.

**Figure 1:**
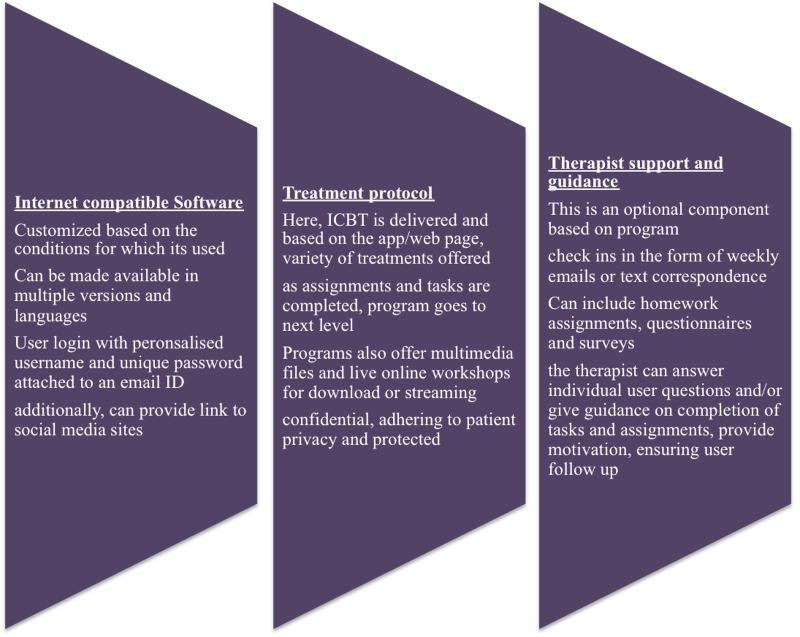
The design of a simple internet-based cognitive behavioral therapy program

The role of the therapist is the least understood component. Paxling identified eight typical therapist behaviors. When therapists encouraged patients’ positive beliefs, showed more empathy, and encouraged the completion of tasks, patients did much better. It resulted in higher rates of task completion by patients [19]. Further work by Schneider and Hadjistavropoulos showed that patients with more severe symptoms prior to treatment, those in psychiatric care, and those currently receiving treatment exhibited better outcomes in response to these behaviors [20-21]. The role of the therapist is still yet to be fully understood and is highly dependent on the users (Figure 1).

Internet-based cognitive behavioral therapy in treatment of psychiatric disorders

According to the World Health Organization (WHO), depression is the third leading cause of disease burden in the world. There have been many advances in treating mental health, but many individuals lack contact with mental health professionals and do not receive help [22]. Patients in treatment for psychiatric disorders are prone to high rates of being lost to follow-up and patient noncompliance. Hence, there is a need for new approaches, connecting technology and medicine. ICBT apps and programs should also include adequate security features to protect patient data, a domain that requires further research [23]. Most ICBT apps include pre- and post-use surveys in the form of questionnaires and also check-ins with the therapist in the form of email or weekly sessions [1]. ICBT can also be used to determine specific risk factors that contribute to psychiatric disorders [24-25]. Furthermore, when compared to ICBT, conventional CBT has been shown to be more time-consuming and expensive with higher dropout rates [26].

ICBT helps provide patients a better understanding of their illness. Especially in cases involving psychosis, ICBT apps have been shown to improve social functioning and lead to better patient knowledge [26]. It is more efficient in managing overall stress, not just in decreasing its severity, but also in reducing urges and stress levels, and increasing cognition and life satisfaction in patients [27]. A similar study was done on gambling in Singapore in which a personalized online feedback survey was developed. Different versions were developed and made available in English and Chinese and demonstrated that the individuals who participated received them well [28].

One of the primary limitations that arose while performing clinical trials on ICBT was the lack of a proper “digital placebo” group [3]. Studies done on ICBT across a wide range of psychiatric conditions shows its effectiveness as an alternative to psychotherapy, but the results differ from user to user. At a self-help program for depression, most patients felt the need for human contact and to speak to a therapist, while patients who were reluctant to accept human contact and talk to a stranger appreciated such a program better [29]. The aspect of writing and doing exercises seemed less important to patients, and the fact that someone was willing to listen motivated them to move towards seeking treatment. The researchers also noted that, like the studies done by Paxling, the patients needed motivation from therapists to do the exercises [19].  

ICBT has also shown considerable efficacy in sleep-related disorders. In the western world, nearly 10% of adults experience insomnia [30]. Cognitive impairment, higher levels of fatigue, and poor concentration, depression, and anxiety are common in those suffering from insomnia [31]. Although several apps exist concerning sleep, Horsch conducted a randomized control trial on the efficacy of ICBT delivered through a sleep app. The advantages of having an app such as this are its portability, since it can be used with any smart device, and its unobtrusiveness. It is also cost saving, as less time with a therapist is needed, and it can achieve high levels of dissemination. The app included a sleep diary, relaxation exercises, and education on sleep hygiene. The app showed considerable effectiveness in dealing with not only insomnia, but also with associated symptoms such as depression and anxiety, and was cost-effective [32].

ICBT has played a significant role in the prevention of suicide. Patients with suicidal thoughts find it harder to seek help and support due to financial factors and social stigma [34]. Though patients benefit from face-to-face therapy, ICBT has shown considerable promise in decreasing suicide attempts, suicidal ideation, and self-harm, especially in the modern-day population [33]. In a similar study, Self-help Online against Suicidal thoughts (SOS), a six-week trial, focused on the intensity and frequency of suicidal thoughts, quality of life of patients, depressive symptoms, healthcare costs, productivity loss, the occurrence of worrying and hopelessness, and secondary outcomes [34]. The results of the study, which is still underway, would show whether internet-based programs for suicide prevention are effective or not. The study also brings to light the need for further research on ICBT and suicide prevention.

Psychiatric disorders are common in chronic and severe medical illnesses. In this section, we describe the effectiveness of ICBT, as shown by various studies, in the treatment of such psychiatric conditions. In diabetes mellitus, one of the most common chronic medical illnesses, depression is twice as common when compared to the general population [35]. Generic programs offering ICBT were found to be efficient and acceptable in patients with diabetes and depression [5]. However, there is a need to tailor programs to those with diabetes mellitus and to further evaluate outcomes in the long-term. Heart failure patients tend to develop more negative thoughts, hopelessness and uncertainty, and anxiety. Because many of them are already taking medications, patients with heart failure prefer psychotherapy. ICBT was time-efficient, with an improvement in depressive symptoms, and was cost-effective [36]. However, we need larger scale randomized control trials to estimate the effectiveness of ICBT in treating heart failure patients based on severity along with a control group.   

During the postpartum period, women experience more stress, anxiety, and symptoms of depression [37]. Pregnant women also tend to use the internet as their predominant source of information [38]. Interventions during the perinatal and postpartum period were found to have a better effect than the ones in the antenatal period [39]. In these women, ICBT resulted in a significant reduction in anxiety, stress, and depression symptoms. In breast cancer survivors, ICBT was shown to improve body image, sexual function, reduce menopause-related symptoms such as hot flashes and sweating, and improved the patients' health and quality of life [9].

Billions of dollars are spent in the United States for pain management, in the form of pain medication. Numerous studies show the benefits of ICBT in managing chronic pain caused by medical illnesses. The annual cost of treatment of pain is more than the cost of treating heart disease, diabetes, and cancer combined. Pain management apps such as e-Ouch or Habit Changer are designed in the form of a pain diary, where patients can log in, make journal entries thrice a day, and get feedback along with coaching. The app also links to social media websites such as Twitter [11]. ICBT has also aided in pain reduction, in improving stiffness, and in reducing distress and symptoms of depression in adults older than 50 years with knee osteoarthritis [40].

The efficacy of ICBT as an established treatment for substance use disorders is not clear. A web-based program determined ICBT to be helpful in decreasing the severity of alcohol consumption [41]. A controlled trial of the program Quit the Shit, done on cannabis users, showed a reduction in marijuana use, its quantity, and frequency. Furthermore, there was also a reduction in depressive and anxiety symptoms and an overall increase in life satisfaction [42]. In veterans with PTSD, depression, and substance use, negative mood regulation (NMR) is a contributing factor for all three problems. Group ICBT treatment resulted in a significant decrease in NMR symptoms, thereby causing a reduction in comorbidities [43].

In critical care, patients undergoing long-term intensive care unit (ICU) treatments, while being exposed to extremely stressful conditions, were found to release higher levels of catecholamines and inflammatory mediators along with experiencing higher levels of pain due to intensive procedures. Such patients and their spouses are at a high risk of developing PTSD, termed post-ICU-PTSD, the symptoms of which were found to reduce the quality of life [44]. In a first of its kind, Gawlytta studied such patients and their spouses and employed internet-based cognitive-behavioral writing therapy (IB-CBWT). The study found that IB-CBWT was effective in this particular cohort of patients. Additionally, It could be easily translated into multiple languages and spread globally [5] (Table 1).

**Table 1:** A table of some of the most commonly used apps in internet-based cognitive behavioral therapy PTSD: Post-traumatic stress syndrome

App/Webpage	Diagnosis used in	About the app/web page
MoodGYM	Depression, anxiety	The structure of the web page is in the form of modules, which have interactive exercises and quizzes. It is self-guided, and only on the completion of a part, the user progresses to the next. User information is protected by confidentiality. Multiple versions of the app are available in Norwegian, Dutch, Chinese, Finnish, and German
MoodHacker	Depression	Mobile-based, self-guided app that promotes “self-management” by improving physical activity, sleep, social support, and nutrition
Living Life to the Full	Anxiety, stress, depression	This web page offers courses in handling daily stress in a self-guided way and trains the user to cope with and address 'unhelpful thinking'. The page also contains free audio tools to deliver content created by psychiatrists for anxiety control, worksheets, and links to social media websites like Facebook and Twitter.
PE Coach	Post-traumatic stress disorder	This app is designed for military veterans and is aimed at managing and treating PTSD through exposure-based treatment. It is therapist-guided and works in conjunction with face-to-face therapy to decrease fear and anxiety symptoms

The cost-effectiveness of ICBT

One of the key concepts to discuss at this point, regarding ICBT, is its cost-effectiveness. The burden of illnesses like depression on the economy ranges to billions of dollars with significant costs in terms of morbidity, patient care, and follow-up. Studies done on the use of ICBT in pediatric OCD patients showed that ICBT had generated societal cost savings up to $144.98 per patient with cost-saving per additional responder being $78 [7]. The study also pointed out the need for further studies on the cost-effectiveness of ICBT and also showed a significant number of quality-adjusted life-years gained per patient [7]. In a similar study done in Spain, the ICBT app “Smiling Is Fun” showed a cost-saving of 169.50 euros per patient and increased both cost-effectiveness and cost utility per patient [6]

Impact of ICBT in the rural United States

ICBT can provide an effective way of delivering mental health therapy to rural communities in the United States. One shortfall of ICBT is that it requires additional training for practitioners that typically work in urban settings and see patients in person. Overcoming the transition from face-to-face care to online treatment can be done with additional training about rural life and the limitations of internet therapy. One option for training such specialists in ICBT is by using online training. Online training can be supplemented to meet many of the functions of face-to-face training and at a lower cost. Bennett-Levy et al. estimate that online training can reduce face-to-face time by at least 50% [45].

According to the literature, people in rural areas are less likely to have access to medical care but are also less likely to have access to an internet connection [46]. The divide between the rural and urban population in terms of online access is due to many key factors such as level of education, income, and the availability of an internet connection. Internet penetration rates are lower in rural areas compared to urban areas, but internet connectivity has increased over the last several years. In 1998, only 28% of Americans in rural areas used the internet. In the year 2000, only 41% of rural residents had online access; but over just a three-year period, rural internet penetration reached 52% by 2003 [47]. This trend has accelerated, and the gap in internet access between rural and the urban United States is closing. In 2015, 69% of rural residents reported using the internet, versus 75% of urban residents [48].

One major limiting factor for providing health care to patients in the rural United States is their increased travel distance. When compared to their urban counterparts, patients from these areas need to travel two to three times further to see medical specialists than those living in urban areas [49]. Over 18% of residents of nonmetropolitan counties suffer from mental illness [50]. ICBT can bring the "office to the patient" rather than the patient going to the hospital. A study consisting 20,693,828 patients found that the median overall one-way travel distance was 7.7 miles to the nearest specialist in the rural area [49]. To further complicate the issue, the most under resourced professionals are mental health care providers [50] (Table 2).

**Table 2:** A table of some key studies of internet-based cognitive behavioral therapy

Author	Year/ Country	Article	Journal	Diagnosis	Findings
Giosan, et al. [1]	2017/England	Reducing depressive symptomatology with a smart phone app: study protocol for a randomized, placebo-controlled trial	Trials	Moderate depression	One of the first active study protocols to involve an active digital placebo group
Kyrios M, et al. [2]	2014/England	Study protocol for a randomized control trial of internet-based cognitive behavioral therapy for obsessive-compulsive disorder	BMC Psychiatry	Obsessive-compulsive disorder	One of the first known trials that is internet-based and therapist-assisted
Oromendia P, et al. [3]	2016/England	Internet-based self-help treatment for panic disorder: a randomized control trial comparing mandatory versus optional complementary psychological support	Cognitive Behavioral Therapy	Panic disorder	The 'scheduled psychological support group' showed lower rates of drop-outs and betters treatment adherence. The second finding was that when the scheduled psychological support was on patient demand, the effect of therapy was poor and dropout rates increased
Newby J, et al. [4]	2017/Canada	Web-based cognitive behavior therapy for depression in people with diabetes mellitus: a randomized control trial	Journal of Medical Internet Research	Major depressive disorder	A benchmark study which showed ICBT’s efficacy in chronic conditions with significant post-treatment changes and decreases in relapse rates
Gawlytta R, et al. [5]	2017/England	Internet-based cognitive behavioral writing therapy for reducing post-traumatic stress after intensive care for sepsis in patients and their spouses (REPAIR): study protocol for a randomized- control trial	BMJ Open	Post-traumatic stress disorder	First randomized control trial which assessed the safety and efficacy of internet-based cognitive writing therapy after sepsis. Also, calls for a ‘psychological placebo control group’ in further such studies
Romero-Sanchiz P, et al. [6]	2017/United States	Economic evaluation of a guided and unguided internet-basedCBT intervention for major depression: results from a multi-center, three-armed randomized controlled trial conducted in primary care	PLoS one	Major depression	First study to evaluate and establish the cost-effectiveness of two ICBT treatments for Spanish patients
Lenhard F, et al. [7]	2017/England	Cost-effectiveness of therapist-guided internet-delivered cognitive behavior therapy for pediatric obsessive-compulsive disorder: results from a randomized controlled trial	BMJ Open	Obsessive-compulsive disorder	Therapist-guided ICBT resulted in significant societal savings
Birney AJ, et al. [10]	2016/Canada	MoodHacker mobile web app with email for adults to eelf-manage mild-to-moderate depression: randomized controlled trial	Journal of Mhealth and Uhealth	Mild-to-moderate depression	The study showed that the effectiveness increased when intervention with counselors was added. The results of the study also showed that the study participants without employee assistance access and low socioeconomic status did poorly
Eckard C, et al. [11]	2016/United States	The integration of technology into treatment programs to aid in reduction of chronic pain	Journal of Pain Management & Medicine	Opioid addiction	ICBT apps when combined with existing pain regimens showed high benefit, but the study also stressed on the need for larger study populations
Christensen H, et al. [13]	2002/Canada	Web-based cognitive behavioral therapy: analysis of site usage and changes in depression and anxiety scores	Journal of Medical Internet research	Major depressive disorder and generalized anxiety disorder	The study showed that the participants with a higher degree of depressive symptoms did better over time compared to low and moderate levels
Noguchi R, et al. [14]	2017/England	Effects of five-minute internet-based cognitive behavioral therapy and simplified emotion-focused mindfulness on depressive symptoms: a randomized controlled trial	BMC Psychiatry	Major depressive disorder	This randomized control trial highlighted some important aspects of how an ICBT program should be structured to maximize benefit. Some factors were that the exercises should be very simple and participants should be given adequate instruction
Calbring P, et al. [15]	2001/Sweden	Treatment of panic disorder via the internet - a randomized trial of a self-help program	Behavioral Therapy	Panic disorder	This randomized control trial showed some important aspects of how an ICBT program should be structured to maximize benefit. Some factors were that the exercises were very simple and participants should be given adequate instruction
Arean PA, et al. [17]	2016/Canada	The use and effectiveness of mobile apps for depression: results from a fully remote clinical trial	Journal of Medical Internet Research	Major depressive disorder	A remote clinical trial on six English-speaking adults with depression using three different self-guided ICBT apps. It was found that the apps had their greatest impact on moderate levels of depression and had an ability to reach many people
Paxling B, et al. [19]	2013/United States	Therapist behaviors in internet-delivered cognitive behavior therapy: analyses of e-mail correspondence in the treatment of generalized anxiety disorder	Behavioral and Cognitive Psychotherapy	Generalized anxiety disorder	The first study in which the contents of therapist emails were analyzed and specific behaviors identified
Schneider LH, et al. [20]	2016/United States	Internet-delivered cognitive behavior therapy for depressive symptoms: an exploratory examination of therapist behaviors and their relationship to outcome and therapeutic alliance	Behavioral and Cognitive Psychotherapy	Major depressive disorder	A systemic examination of the eight therapist behaviors identified by Paxling et al. in his study and observation of their generalizability
Hadjistavropoulos HD, et al. [21]	2016/England	Predicting response to therapist-assisted internet-delivered cognitive behavior therapy for depression or anxiety within an open dissemination trial	Behavior Therapy	Major depressive disorder and generalized anxiety disorder	A study on 195 patients who were offered 12 modules of therapist-guided ICBT for depression and generalized anxiety, to predict response to therapy. Starting fewer modules were associated with more therapist phone calls, completion of more modules and greater severity before treatment was associated with higher benefits of ICBT

Limitations

ICBT is not exempt from limitations. Many studies show a lack of a placebo group in determining the effectiveness of ICBT and almost all studies discuss the need for larger, randomized controlled trials. Regarding the therapist, training has to be given, and their role needs to be explored, to ensure follow-up with patients and their compliance. Another important aspect is the need for practically constructed apps and web pages with stable software and adequate security measures to protect patient information. While patients benefit from such internet-based interventions, a significant number prefer face-to-face CBT sessions with a therapist. Furthermore, there is a clear need for larger scale studies to establish the effectiveness of ICBT as an alternative psychotherapy tool. Finally, there are ethical, social, and cultural aspects to consider along with the possible short- and long-term side-effects which may occur with the use of ICBT.

## Conclusions

In this literature review, the effectiveness of ICBT was explored. Findings showed that ICBT is effective in the treatment and management of various psychiatric disorders such as depression, GAD and social anxiety, panic disorders, phobias, addiction and substance use disorders, adjustment disorder, bipolar disorder, and OCD. ICBT has been effective in managing the comorbid mental health conditions of medical illnesses such as depression in patients with diabetes mellitus. Moreover, it is cost-effective, both for patients regarding consultation fees and also for the health care system regarding controlling the cost burden.

In rural United States, a considerable lack of access to healthcare is seen, making ICBT an effective alternative. Furthermore, internet access has increased in these areas, and studies need to be done on the level of penetration of ICBT here. Patients can be educated on the transition from face-to-face therapy to internet-based therapy, and the therapists can undergo online training in ICBT. Overall, ICBT is an effective means of treating various mental health illnesses and improving patient care while being cost-effective. Further studies on the use of ICBT in everyday clinical practice, used in conjunction with face-to-face psychotherapy, is of prime importance.
